# Lifeworld-led research: a phenomenological approach to grant experts by experience in vulnerable positions their right to participate in healthcare research

**DOI:** 10.1080/17482631.2025.2522875

**Published:** 2025-06-26

**Authors:** Elvira Pértega, Christopher Holmberg, Karin Dahlberg, Helena Dahlberg

**Affiliations:** a Faculty of Law, University of Technology Sydney, Sydney, Australia; b Department of Psychotic Disorders, Sahlgrenska University Hospital, Gothenburg, Sweden; c Faculty of Health and Life Sciences, Linnaeus University, Växjö, Sweden; d Institute of Health and Care Sciences, University of Gothenburg, Göteborgs Universitet, Gothenburg, Växjö, Sweden

**Keywords:** Human rights, lifeworld research, patient participation, phenomenology, research methodology, vulnerable populations

## Abstract

**Purpose:**

We advocate for a phenomenological lifeworld-led research approach that provides ontological and epistemological foundations for an open methodology that emphasizes the in-depth exploration of human phenomena. This approach invites multiple perspectives to facilitate meaningful, equitable participation of ‘experts by experience.’ We caution against superficially designating participants as “co-researchers,” as this might obscure underlying power imbalances.

**Methods:**

Drawing from Husserl, Merleau-Ponty, and Gadamer, we outline a theoretical framework for lifeworld-led research. This methodology prioritizes open-ended data collection methods, including interviews, observations, written narratives, and supplementary approaches (visual tools, dialogue cards). Reflective, rigorous meaning analysis—rather than content analysis—guides interpretation to capture the complexity of participants lived experiences.

**Results:**

This methodology requires adopting an attitude of open awareness, described as a “bridled” attitude, whereby researchers deliberately slow their understanding to avoid prematurely drawing conclusions. While universal meanings (meaningfulness, love, respect) may be shared, individuals uniquely experience these phenomena. Rather than homogenizing patient groups, we recognize that patients with similar diagnoses may share experiences while maintaining distinct, individual insights.

**Discussion:**

Our approach clearly delineates the roles of professional researchers’ and ‘experts by experience.’ The lifeworld-led approach utilizes participants as “experts by experience,” explicitly acknowledging inherent contradictions and complexities while avoiding overpowering their voices.

## Introduction

Global health research is increasingly integrating collaboration between researchers and stakeholders, including healthcare professionals and patients, to co-produce and apply knowledge (Zych et al., 2020). This practice is actively encouraged by journal policies and funding guidelines (Frisch et al., 2020; Price et al., 2018). Known by various terms—like patient and public involvement (PPI), co-research, and co-design (Slattery et al., 2020)—these approaches emphasize research “with” or “by” patients, rather than “for” or “about” them, underpinned by ethical, political, and methodological motivations (Lefkowitz et al., 2022; Ward et al., 2010). This shift promotes equity by aligning research goals with community priorities (Lefkowitz et al., 2022).

While PPI offers valuable insights, certain challenges remain underexplored, such as compensation, confidentiality, and risks of tokenism—where patients may be superficially included to satisfy external agendas without meaningful engagement (Majid, 2020; Ward et al., 2010). There may be ethical and methodological issues when positioning patients as co-researchers: it is vital that move beyond logistical considerations when addressing this issue. A key concern, central to the argument in this paper, is that some research arrangements, such as the ’co-research approach’ when understood primarily as task-oriented research collaboration, might not properly acknowledge, value or ultimately respect participants’ specific worldviews and unique experiences. We refer to a participatory research practice extending beyond a fragmented PPI limited to certain stages or duties of the research process, towards as a deeper way to make use of the expertise of people with lived experience of the phenomenon being explored. A phenomenological lifeworld-led approach is more suitable to enable acknowledgement and inclusion of participants’ lived experiences, emphasizing the uniqueness of their existential reality rather than imposing representational obligations. This supports more ethical research practices by allowing participants to freely share the knowledge born of their experiences, rather than forcing participants to represent a wider group. Although our focus is on patients in health care, we also emphasize how such an approach reduces the exclusion of marginalized populations by asking participants unconditionally to share insights based on personal experience, instead of on sociodemographic characteristics as representatives of a broader group. By doing so we hope to address the “representative fallacy”, which happens when researchers wrongly assume that the experience of one patient represents everyone who has the same condition, thereby diminishing the value of singular experiences. In health care settings this runs the risk of committing epistemic injustice (the unfair treatment of individuals’ knowledge due to lack of acknowledgement). This is crucial, as assuming sharing similar sociodemographic characteristics or certain health condition means certain patients can speak for others ignores the uniqueness of human experiences and risks obscuring the power imbalances and neglects how diversity shapes illness narratives (Lefkowitz et al., 2022).

The lifeworld-led research approach argues that patients must not be considered as spokespersons, rather than their insights—much like those offered by a clinician in the professional area—must be given status of expertise in the patient field in virtue of how much their knowledge informs what research is relevant and ethically and methodologically sound to their needs and interests, among many other crucial ways, as shall be clarified. Instead of viewing patients as spokespersons, their involvement should highlight their expertise in navigating illness, akin to clinicians’ roles, thus enhancing ethical, substantive engagement in research.

### The right to participate in scientific research

A human rights approach to science ensures that research is conducted with a commitment to core human rights principles: participation, empowerment, non-discrimination, and accountability (Arstein-Kerslake et al., 2019). Of specific interest is the importance of guaranteeing truly inclusive settings where participants feel valued and respected. The pitfalls of “tokenism” where individuals are considered “co-researchers” (Majid, 2020) when assigned certain research tasks can sometimes make them fall short in delivering proper participatory practice. While assigning participants to co-research positions is frequently done with equality in mind, it is often more an illusory demonstration of power balance than truly effective inclusive practice. This is the case as it is the researcher in the first place who is in power to decide the level and quality of PPI. To overcome this power differential, the primary concern should be finding ways of meaningful PPI (Boyle & Stew, 2025) that mirror worldview and lived experience, as this is a clear indicator of how power and privilege are distributed in health care (Majid, 2020; Ward et al., 2010).

As argued by the Special Rapporteur in the field of cultural rights (Xanthaki, 2024), authentic participation requires “context-sensitivity” that reflects individuals’ identities, values and experiences. In this sense, methodologies that empower marginalized groups should seek to create authentic transformative roles rather than superficial involvement. A human rights-based approach to research provides a solid ethical and philosophical foundation to establish a method for projects involving participants in vulnerable situations, like populations lacking social, political and financial stability, children, people living with physical, psychological or cognitive impairment.

However, methodologies developed to grant the right to participate in science such as emancipatory, participatory, and, more recently, human rights-based methodology face limitations. Involving participants as co-researchers diminish the authenticity of their contributions by assuming equal responsibilities without equal preparation or support, not clarifying how to democratize the co-produced research process (Liddiard et al., 2019). Also of special relevance for this paper’s approach, are considerations regarding how assessment of competence and capacity may prevent traditionally marginalized populations from fully engaging with scientific knowledge in the research process. It is precisely these kinds of difficulties that justify this paper’s main goal: establishing phenomenological methodology as suitable, human rights approach that acknowledges that certified expertise and experiential expertise bring different—but complementary—forms of knowledge and skills (Promotion and protection of all human rights, n.d.). Thus, instead of considering that meaningful PPI contributes to making phenomenological research more credible (Boyle & Stew, 2025), we argue that it is the phenomenological approach that contributes to making PPI truly meaningful. All must have equal access to substantial ways of producing and transforming our societies using the right to participate in scientific advancement to make changes in accordance with specific realities and ways of living as well as for diverse settings and identities. For instance, recognizing the patient perspective implies understanding that people do not have to act like researchers to contribute meaningfully to scientific knowledge in our social context, thus avoiding tokenism and providing an accurate recognition of “experiential” expertise (Promotion and protection of all human rights, n.d.).

As noted by Murris (2013), there is a core “epistemic challenge” (related to how knowledge is constructed, acquired, and legitimized in different settings and power structures): how to appreciate the capacity of the knowledge of research subjects while simultaneously making explicit how their views are considered and valued in actual scientific processes. The main theoretical proposal of this article—that a phenomenological method for conducting health research which positions patients and participants as “experts by experience”—directly responds to this need in practical terms.

### A phenomenological understanding of the right to participate in research

With their analyses in the nineteenth and twentieth centuries of the human existence and the lived experience, the philosophies of Husserl (1970a, 1970b), Merleau-Ponty (1995), and Gadamer (1976) offer science a radically new way of understanding humans and how we relate to our world. Different from the contemporary positivist philosophy of science, which treats humans on the one hand and the world on the other, separating them and describing them as reduced parts, phenomenology explicitly explains how individuals always find themselves as beings in and of the world. There is an essential in-between, where meaning is born. Existence cannot be reduced to a least common denominator or polarized parts but must be understood in its complexities of meaning.

In particular, phenomenology displays the intentional relationship between humans and the world and how meaning arises from this interaction. In this way, phenomenology provides a rich ontological, epistemological, and methodological foundation that is inherently suitable for ensuring that research grants the right to meaningful participation. Unlike approaches that emphasize roles such as “co-researchers,” that can blur boundaries sometimes resulting in tokenism, phenomenological research focuses on participants as “experts by experience” (Lefkowitz et al., 2022). This allows participants to share insights authentically and without expectations of formal research training. It fosters meaningful engagement that honours their lived reality, focusing on the depth and complexity of human experience rather than assigning tasks that may not be appropriate or relevant for the participants.

A phenomenological approach to research offers a new way to consider participation in research where subjectivity, competency, and capacity are understood as relational and situational qualities (Tisdall, 2018). By adopting this approach, we align with broader discussions about involving patients in research, where participation is encouraged based on collaborative relationships that facilitate a deeper exploration of the lived expertise rather than on assessing individual capacity to accordingly assign certain research tasks.

### Lifeworld-led research

Phenomenology emphasizes how human phenomena must be understood in their complexity and context, employing scientifically accepted methods that adapt to the specific needs of participants and their situations. Placing lived experience at the forefront supports the right to participate in science, allowing participants to express what is most meaningful in their life contexts (Dahlberg et al., 2008; Frisch et al., 2020). This ensures that participants’ voices are authentically represented, thus addressing the epistemic injustices marginalized groups often face (Fricker, 2007; Murris, 2013).

We offer an approach of lifeworld-led research as an alternative strategy to task-oriented co-researching, having the advantage of more profound and meaningful participation over other more shallow approaches to patient involvement in research. The lifeworld theory is at the core of phenomenology and phenomenological research (Dahlberg et al., 2008). Here, we find arguments to re-visit the question of patient involvement and meaningful participation in health care research, or research that involves marginalized groups. Lifeworld-led research explores the experiential horizons that provide us with individual and unique meaning. Such research is open to meaning of what the participants have experienced and have the ability to express, and recognizes the vulnerability that may exist when, for example, in need of help from professional experts in health care.

The lifeworld is unique to every individual, but is not a bubble that isolates a person from others’ experiential domains; we are also part of and share the same world and social existence (Dahlberg et al., 2009; Todres et al., 2007). We share universal meanings such as the desire for meaningfulness, togetherness, love, and respect. We may also share language and culture with others that may open for deeper understanding. And it is not about either uniqueness or sameness: the uniqueness of every human being sets the tone in the way that *the how* of the sameness varies. Even if we share the same aspects of existence, we may experience them differently. Patients, for instance, do not represent a homogeneous group but are individuals who may share some experiences with other patients with the same diagnosis but may also have distinctive experiences. This is why the lifeworld theory is essential also to advanced health care research that benefits from the clear roles of professional researchers and patients who are experts in and of their own lives.

An important aspect of the lifeworld is our everyday life, where we sense, feel, think, and dream, etc. Lifeworld-led philosophy acknowledges how humans perceive the world in one’s own and habitual way but is also directed at the goal of the activity. Merleau-Ponty (1995) gives us an example of a blind man with his cane. He is walking along the road, and his attention is to where he is going, not what he has in his hands. The cane for him is not a thing, it is instead something that he is *perceiving with* rather than *perceiving at*. The blind man extends his body to involve the cane. Merleau-Ponty expresses human perception and emphasizes how we are always directed at something, involving the intention of our activities, always in a relationship with the world.

Merleau-Ponty (1995) addresses the lifeworld as the world of perception: “the world which is revealed to us by our senses and in everyday life.” This world, he says, “seems at first sight to be the one we know best of all. We need neither to measure nor to calculate to gain access to this world, and it would seem that we can fathom it simply by opening our eyes and getting on with our lives. Yet this is a delusion”. The lifeworld is “to a great extent, unknown territory as long as we remain in the practical or utilitarian attitude” (p. 39).

As a philosopher, Merleau-Ponty is not only *in* the lifeworld, but also looking *at* the lifeworld. He gives us the understanding of *how* we perceive everything that we encounter in everyday life as well as in research. His philosophy can teach researchers to be aware of how our participants perceive some phenomena and situations of interest, but also how we as researchers must be more like the philosopher, i.e., be part of the lifeworld and at the same time also exploring it (H. Dahlberg, 2022). It is an epistemic demand that researchers have the schooling and insights of how to change their attitude, i.e., how to escape/avoid the everyday, practical, and utilitarian stance where one takes for granted that what one grasps with one’s senses *is* what it seems to be, in favour of a scientific attitude of alert openness where one dwells with and examines the participants’ experience. Through such an attitude the researcher can not only listen carefully to participants’ experiential descriptions, but also see more and deeper meaning of them. In understanding that participants’ epistemological stance and expertise is different from the researchers’, the potential for meaningful PPI is not diminished at the same time that methodological rigour is not jeopardized (Boyle & Stew, 2025).

The change of attitude that is needed must be characterized by a form of open awareness that previously has been described as a “bridled” attitude, where researchers, in a thoughtful way, slow down their process of understanding instead of hastily jumping to a conclusion about what one perceives or understands (Dahlberg, 2019; H. Dahlberg & Dahlberg, 2019, 2020; K. Dahlberg et al., 2008). Through bridling, the researcher becomes a medium by which the participant’s message is clarified and strengthened.

For study participants, the approach is different. The role of “co-researcher” might give the impression that they should adopt a scientific perspective and bridled attitude, which is a poor solution. They should instead be encouraged to remember and reflect on their personal and unique experiences in daily life, what it means for them to live with illness, the significance of vulnerability, and/or the need for care. We want to focus on their everyday life and existence, as they live, relate to and perceive what they encounter, without any restrictions that belong only to the researchers.

### How to involve patients as experiential participants

A phenomenological research approach, such as Reflective Lifeworld Research (Dahlberg 2019); H. Dahlberg et al. 2024); Dahlberg and Dahlberg 2020); Dahlberg et al. 2008)), has full capacity to encounter participants and their lived experience, standing up for them in a way that asks experiential questions from them, listening to them when they want to describe how it is and what it, for example, means to be ill and receiving care. The essential role of the lifeworld-led researcher is even more than listening carefully; it is to use one’s scientific knowledge and experience to describe the meaning that arises from the investigation professionally and thoroughly. Such an approach is accomplished by careful choices of methods, both for data generation and data analysis. There are no pre-determined methods or steps; all lifeworld-led research is phenomenon-oriented and methods for data generation and analysis are carefully chosen and practiced. Interviews, observations, and written accounts are common in lifeworld-led research for their sensitivity to existential phenomena. A study’s design can include one or several methods, each supported by various means. Interviews or observations may, for example, be supplemented with means such as drawings, dialogue cards, and video, to satisfactorily clarify the phenomenon. In some contexts, narratives, e.g., diary keeping may be good extra sources. Still, it is not the amount of data that matters most. In qualitative, lifeworld-led research, what truly counts is that the participants are given the right time and space so that the data are meaningful, rather than merely plentiful (Jared, 2023).

For the data analysis, meaning must be in focus. To note content, i.e., what the participants convey, is not enough (Dahlberg et al., 2024). Meaning analysis reveals contradictions or other weaknesses in data, which would be omitted by content analysis. Meaning analysis is tailored to deal with how the same expressions may reveal different meanings or how different expressions reveal the same meaning. Such complexities must be possible to disclose and communicate competently if the aim is to give voice to vulnerable persons or marginalized groups of people.

### Addressing the problem of representative fallacy

Phenomenology also addresses the “representative fallacy,” which reproduces the epistemic injustice of assuming that those being listened to are entitled to speak on behalf of others with whom they share a similar characteristic, in health care contexts it is usually a particular condition or disease. This might disregard uniqueness and reinforce power imbalances within marginalized groups, as certain patients are perceived to represent all persons with a certain illness. This also neglects intersectionality, such that patients with the same illnesses do have variations in their socioeconomic status and different possibilities for practicing self-care (Lefkowitz et al., 2022).

Representing such diversity, especially among specific demographic groups such as ethnic minorities, is a complex issue. It can isolate a person’s lived experience from the cultural, socioeconomic, and other factors that shape their reality. Sometimes, certain phenomena may demand extra attention. As Vagle (2018) suggests, phenomena should be approached as if they are always in the process of being formed and re-formed by numerous forces—both human and non-human. Vagle (2018) reminds us that this perspective has long been present in the theorizing and knowledge traditions of Black, Indigenous, and People of Color (BIPOC). With this awareness, researchers—particularly those who are privileged, i.e., male and white—must think more broadly and creatively about what constitutes the data they work with. According to Vagle (2018), phenomenological research calls for exploring diverse materials to see how they might prompt new understandings or perspectives. Such materials may include histories of oppression, individuals’ own narratives and discourses, their spaces and contexts, relevant policies, and even philosophical concepts. These can all serve as phenomenological material, complementing the patient’s lived experiences.

Vagle’s (2018) ideas further stress our argument to view researchers as experts in leveraging their methodological expertise, while participants provide unique insights that enhance the depth and authenticity of findings. Just as researchers do not claim to represent other researchers but instead rely on established methods and knowledge within their field, patients should be similarly recognized for the valuable insights they provide. Their expert knowledge of living with an illness should not be reduced to shallow symbolism.

### Further participant involvement

Participants can be included on different occasions in a study. As Halling et al. (2006) suggested, phenomenological research can be dialogic. As these researchers have experienced, projects benefit from dialogical meetings to plan, define aims and goals, discuss methods as well as reflect on results. In their practice, the dialogical meetings have invited other researchers and/or research students, but this approach can be practiced to involve for example patients and students. Persons with experience of certain illnesses and/or professional care of particular interest for a study can be invited to participate in all levels of a phenomenological study. It is essential that they are invited as laypeople who are experts on themselves and their own illness trajectory and not as experts on other patients or as healthcare professionals or research professionals. In this way, this form of participant involvement completes the lifeworld-led research and optimizes a person-centred approach, benefitting from expertise in different areas.

By employing the most suitable method for data generation, together with a meaning-oriented analysis, researchers can gain a comprehensive understanding of the patient’s lifeworld, capturing the multifaceted nature of their healthcare experiences. This approach allows for a more holistic view of patient care, encompassing temporality, such as how patients experience time during their healthcare journey, spatiality, and the impact of physical environments on their experiences. It could also lead to a better understanding of embodiment, how patients perceive and relate to their bodies during illness or treatment, and sociality, the meaning of relationships, and social interactions during healthcare experiences (Todres et al., 2007).

## Conclusion

Despite their different attributes, all people must be listened to, including those with disabilities, dementia, mental disorders, and other illnesses, as well as women, men, children, people with “different” religion, culture, or other qualities that are more or less unseen or not fully respected. This should be the number one priority for all researchers. Phenomenology and lifeworld-led research contribute a rich ontological, epistemological, and methodological foundation, already suited to include existential matters, however complex and multifaceted these phenomena may be. A phenomenological approach investigates, explores, describes, interprets, and explains phenomena that are difficult or impossible to measure. In lifeworld-led investigations, researchers seek to strengthen the voice of participants and look for the meaning in their expressions.

However, the participants can never be understood as researchers. If the goal is to produce an evident research outcome, it is crucial to benefit from all partners’ areas of expertise. Researchers are expected to be experts in science and research, including the theory of science. For participants, it is to be experts on what it means, for example, to live with a disease, illness, or injury, or to belong to a marginalized group of people, wanting to find meaning in life, experiencing well-being, and being able to fulfill once desired goals and wishes.

Phenomenology, with its philosophical depth and adaptability, can add to the already available methodologies that enhance a human rights approach to science (such as emancipatory, participatory, and, more recently, human rights-based methodology) by facilitating meaningful participant engagement without requiring participants to take on formal co-researcher roles. By recognizing participants as “experts by experience” instead of assigning them certain research tasks, we acknowledge their vital contribution to understanding the phenomena being studied, while avoiding the illusion of becoming a trained researcher only through one’s lived experiences. Clearly defining the roles of both researchers and participants fosters a collaborative partnership that maintains distinct boundaries and complementary potential. This approach combines the methodological expertise of researchers with the unique perspectives of participants, resulting in research outcomes that are both authentic and comprehensive.

## Data Availability

Data sharing is not applicable to this article as no new data were created or analysed in this study.
